# Supplementary material for: Pathways of topological rank analysis (PoTRA): a novel method to detect pathways involved in hepatocellular carcinoma
Source: PeerJ. 2018 Apr 9;6:e4571. doi: 10.7717/peerj.4571 (PMC5896492; doi:10.7717/peerj.4571)
Supplement: Table S1 — We consolidate the information from Section 1.1–Section 1.2 into one table with four columns, namely: (1) Gene index (1–250) (2) Gene symbol (3) The PageRank score of each gene in this pathway for normal (4) The PageRank score of each gene in this pathway for cancer. (5) The variance of gene expression for each gene across normal samples. (6) The variance of gene expression for each gene across cancer samples. [file peerj-06-4571-s001.docx]

| Index | Gene Symbol | PR.normal | PR.case | var.normal | var.cancer |
| --- | --- | --- | --- | --- | --- |
| 1 | AKT3 | 0.003751 | 0.002902 | 16449.11 | 90046.23 |
| 2 | RASGRP1 | 0.003815 | 0.003484 | 8219.892 | 23022.37 |
| 3 | RASGRP2 | 0.005625 | 0.005522 | 17092.59 | 28902.84 |
| 4 | CACNG3 | 0.002421 | 0.003029 | 0.385306 | 0.300408 |
| 5 | CACNG2 | 0.000623 | 0.001776 | 0 | 1.732245 |
| 6 | TAB1 | 0.004338 | 0.005458 | 53969.27 | 376577.9 |
| 7 | GADD45G | 0.004646 | 0.001976 | 10303884 | 11316190 |
| 8 | MAP4K1 | 0.003759 | 0.006678 | 32916.26 | 66615.52 |
| 9 | DUSP10 | 0.004424 | 0.001447 | 2633702 | 2737817 |
| 10 | CHUK | 0.004585 | 0.004084 | 59221.94 | 249851.1 |
| 11 | RASGRP4 | 0.003905 | 0.006033 | 1340.947 | 1136.418 |
| 12 | MAP3K8 | 0.00448 | 0.003726 | 13189.54 | 210032.8 |
| 13 | MAPK14 | 0.004111 | 0.004148 | 459233 | 2452508 |
| 14 | DAXX | 0.004153 | 0.005471 | 266675.1 | 1474341 |
| 15 | GADD45A | 0.004384 | 0.004395 | 4331451 | 5098307 |
| 16 | DUSP1 | 0.006048 | 0.002719 | 1.62E+08 | 1.47E+08 |
| 17 | DUSP2 | 0.005023 | 0.003304 | 66859.33 | 108604.4 |
| 18 | DUSP3 | 0.004196 | 0.007498 | 1844063 | 13272189 |
| 19 | DUSP4 | 0.004941 | 0.002783 | 6326.847 | 438770.5 |
| 20 | DUSP5 | 0.005806 | 0.003232 | 2474273 | 1096331 |
| 21 | DUSP6 | 0.004833 | 0.005301 | 22008653 | 7587178 |
| 22 | DUSP7 | 0.003071 | 0.005592 | 13804.62 | 131069.5 |
| 23 | DUSP8 | 0.005179 | 0.004652 | 162070.9 | 247835.2 |
| 24 | DUSP9 | 0.00516 | 0.004515 | 180.8086 | 7901776 |
| 25 | EGF | 0.001868 | 0.00208 | 2.142449 | 12572.82 |
| 26 | ELK1 | 0.003804 | 0.00444 | 106272.1 | 327025.8 |
| 27 | ELK4 | 0.004895 | 0.005496 | 5403.498 | 22862.79 |
| 28 | AKT1 | 0.0062 | 0.001187 | 1914981 | 8615938 |
| 29 | AKT2 | 0.004386 | 0.003586 | 1474931 | 3520700 |
| 30 | MECOM | 0.00491 | 0.006745 | 3571.806 | 110421 |
| 31 | FGF1 | 0.005415 | 0.002466 | 722.9812 | 105680.2 |
| 32 | FGF2 | 0.005091 | 0.002817 | 14932.03 | 138053.3 |
| 33 | FGF3 | 0.000623 | 0.001888 | 0 | 19980.87 |
| 34 | FGF4 | 0.000623 | 0.000981 | 0 | 32.73306 |
| 35 | FGF5 | 0.002225 | 0.001965 | 1.773061 | 4.703673 |
| 36 | FGF6 | 0.000623 | 0.000608 | 0 | 0 |
| 37 | FGF7 | 0.004601 | 0.003112 | 749.8106 | 10832.72 |
| 38 | FGF8 | 0.001339 | 0.001462 | 0.338367 | 44.13714 |
| 39 | FGF9 | 0.005379 | 0.006768 | 4.428163 | 68.95673 |
| 40 | FGF10 | 0.002796 | 0.0038 | 0.548571 | 0.098367 |
| 41 | FGF11 | 0.00388 | 0.005846 | 183.68 | 421.6914 |
| 42 | FGF12 | 0.003921 | 0.002462 | 2460.923 | 16122.6 |
| 43 | FGF13 | 0.004873 | 0.002215 | 2730.123 | 1002058 |
| 44 | FGF14 | 0.000808 | 0.002307 | 48972.16 | 203103.7 |
| 45 | RRAS2 | 0.004662 | 0.005531 | 61568.12 | 241163.3 |
| 46 | MRAS | 0.003885 | 0.002968 | 31471.52 | 964275.1 |
| 47 | TAB2 | 0.00346 | 0.005479 | 824975.7 | 4030567 |
| 48 | MAPK8IP3 | 0.005186 | 0.004073 | 46581.59 | 268541.7 |
| 49 | MAPK8IP2 | 0.003886 | 0.002789 | 1547.549 | 130858.1 |
| 50 | RASGRP3 | 0.004851 | 0.008414 | 36394.99 | 108816.8 |
| 51 | FGF20 | 0.001324 | 0.001889 | 0.267755 | 2309.51 |
| 52 | FGF21 | 0.004972 | 0.005344 | 3867185 | 6600275 |
| 53 | FGF22 | 0.000623 | 0.001324 | 0.277959 | 13.56571 |
| 54 | CACNG5 | 0.000623 | 0.001009 | 0 | 0.02 |
| 55 | CACNG4 | 0.002059 | 0.001914 | 320.8086 | 16833.8 |
| 56 | RPS6KA6 | 0.005964 | 0.002111 | 32.44449 | 130.8282 |
| 57 | GNA12 | 0.004317 | 0.007287 | 94479.95 | 825683.5 |
| 58 | MKNK2 | 0.005303 | 0.007579 | 1379414 | 12965859 |
| 59 | GRB2 | 0.002362 | 0.005664 | 842807.1 | 4904509 |
| 60 | HRAS | 0.00435 | 0.003362 | 19500.81 | 1083630 |
| 61 | HSPA1A | 0.001742 | 0.004197 | 1.66E+08 | 6.25E+08 |
| 62 | HSPA1B | 0.002462 | 0.001729 | 5139311 | 4823973 |
| 63 | HSPA1L | 0.006201 | 0.002373 | 257.5759 | 4938.343 |
| 64 | HSPA2 | 0.004604 | 0.004506 | 39587.76 | 109769.7 |
| 65 | HSPA6 | 0.004272 | 0.002822 | 85354.74 | 347373.6 |
| 66 | HSPA8 | 0.003807 | 0.007458 | 1.05E+08 | 6.25E+08 |
| 67 | FAS | 0.00407 | 0.003714 | 152130 | 534800.7 |
| 68 | IKBKB | 0.003185 | 0.008582 | 70127.19 | 249619.7 |
| 69 | IL1A | 0.006069 | 0.002261 | 2.999592 | 2.000408 |
| 70 | IL1B | 0.007113 | 0.005098 | 32031.1 | 5460.033 |
| 71 | IL1R1 | 0.004445 | 0.005354 | 3691136 | 13476831 |
| 72 | FASLG | 0.004121 | 0.005201 | 604.5229 | 6861.918 |
| 73 | KRAS | 0.003372 | 0.004911 | 176723.6 | 415590.4 |
| 74 | MAP3K1 | 0.003485 | 0.005704 | 67903.03 | 146670.5 |
| 75 | MAP3K5 | 0.00361 | 0.001555 | 146049.3 | 1068973 |
| 76 | MAP3K11 | 0.007389 | 0.005482 | 696330.2 | 7048707 |
| 77 | MOS | 0.001575 | 0.000608 | 0.02 | 0 |
| 78 | GADD45B | 0.002584 | 0.002872 | 98071769 | 33556534 |
| 79 | NF1 | 0.003677 | 0.004086 | 327634.2 | 1247073 |
| 80 | NGF | 0.005933 | 0.00271 | 983.5086 | 866.2143 |
| 81 | NRAS | 0.003958 | 0.005077 | 221410.4 | 3108954 |
| 82 | NTF3 | 0.006554 | 0.002633 | 2707.968 | 1966.123 |
| 83 | NTF4 | 0.001424 | 0.002221 | 0.297551 | 0.232245 |
| 84 | PAK1 | 0.004128 | 0.00638 | 102089.3 | 1007648 |
| 85 | PAK2 | 0.003617 | 0.006467 | 1033319 | 3079869 |
| 86 | ECSIT | 0.003912 | 0.002213 | 311537.2 | 1189097 |
| 87 | PDGFA | 0.00575 | 0.002691 | 32155.28 | 4384435 |
| 88 | PDGFB | 0.005627 | 0.005115 | 46156.06 | 311469.6 |
| 89 | MAP3K20 | 0.005016 | 0.003901 | 434277.7 | 2819078 |
| 90 | PPM1A | 0.004475 | 0.004707 | 669292.3 | 1203681 |
| 91 | PPM1B | 0.004199 | 0.004669 | 340902.9 | 1075666 |
| 92 | PPP3CA | 0.005373 | 0.007287 | 104116.8 | 172458.6 |
| 93 | PPP3CB | 0.003364 | 0.005843 | 75297.65 | 314164.4 |
| 94 | PPP3CC | 0.003668 | 0.010776 | 15208.33 | 31096.69 |
| 95 | PPP3R1 | 0.00463 | 0.005404 | 462072 | 1389662 |
| 96 | PPP3R2 | 0.002953 | 0.00218 | 0.163673 | 4.132653 |
| 97 | PPP5C | 0.00237 | 0.007656 | 162601.2 | 453638.7 |
| 98 | PRKACA | 0.00417 | 0.003334 | 1224766 | 29880755 |
| 99 | PRKACB | 0.00364 | 0.005145 | 141808.1 | 409270.4 |
| 100 | PRKACG | 0.00097 | 0.000608 | 0.107755 | 0.244898 |
| 101 | PRKCA | 0.005571 | 0.00613 | 100625.8 | 2142287 |
| 102 | PRKCB | 0.004089 | 0.003235 | 56204.51 | 92345.06 |
| 103 | CACNA2D3 | 0.006015 | 0.001517 | 14.53714 | 94294.34 |
| 104 | PRKCG | 0.002665 | 0.000608 | 0.942449 | 2.204082 |
| 105 | MAPK1 | 0.003744 | 0.003396 | 790412.6 | 4719162 |
| 106 | MAPK3 | 0.003447 | 0.005165 | 45893.81 | 655005 |
| 107 | GNG12 | 0.003954 | 0.00488 | 715806.6 | 3369262 |
| 108 | MAPK7 | 0.003476 | 0.007928 | 5060.738 | 29434.07 |
| 109 | MAPK8 | 0.003345 | 0.003471 | 17322.39 | 55936.17 |
| 110 | MAPK11 | 0.004969 | 0.002878 | 4647.827 | 70452.8 |
| 111 | MAPK9 | 0.004332 | 0.005686 | 70459.52 | 521973.4 |
| 112 | MAPK10 | 0.004552 | 0.002474 | 581.231 | 3696.939 |
| 113 | MAPK13 | 0.00408 | 0.004086 | 19409.69 | 299547.8 |
| 114 | MAP2K1 | 0.003102 | 0.006487 | 1412027 | 725426.6 |
| 115 | MAP2K2 | 0.004257 | 0.00247 | 468312.1 | 8694997 |
| 116 | MAP2K3 | 0.002959 | 0.001456 | 5884543 | 1423073 |
| 117 | MAP2K5 | 0.00471 | 0.006529 | 12510.11 | 39251.77 |
| 118 | MAP2K6 | 0.004774 | 0.001076 | 16020.45 | 975654.4 |
| 119 | MAP2K7 | 0.005538 | 0.005056 | 88167.27 | 513222 |
| 120 | PTPN7 | 0.003478 | 0.004637 | 28419.77 | 102580.7 |
| 121 | PTPRR | 0.004283 | 0.000858 | 11.91388 | 162.7037 |
| 122 | RAC1 | 0.004104 | 0.004701 | 2401274 | 14310764 |
| 123 | RAC2 | 0.004743 | 0.004455 | 265361.4 | 1158529 |
| 124 | RAC3 | 0.004445 | 0.002612 | 16239.48 | 628662.1 |
| 125 | RAF1 | 0.004236 | 0.005353 | 866327.9 | 7042571 |
| 126 | RAP1A | 0.004877 | 0.003717 | 425437.5 | 907014.1 |
| 127 | RAP1B | 0.005106 | 0.006776 | 597767.7 | 737574.9 |
| 128 | RASA1 | 0.003204 | 0.006094 | 38566.55 | 177339.3 |
| 129 | RASA2 | 0.004744 | 0.007148 | 2036.172 | 4369.596 |
| 130 | RASGRF1 | 0.003481 | 0.001698 | 39.50367 | 1536056 |
| 131 | RASGRF2 | 0.005265 | 0.004644 | 263.6004 | 24325.31 |
| 132 | CACNG8 | 0.000623 | 0.00423 | 0 | 0.256735 |
| 133 | CACNG7 | 0.000623 | 0.001632 | 0.400408 | 2.901633 |
| 134 | CACNG6 | 0.004696 | 0.00104 | 0.575102 | 0.338367 |
| 135 | RPS6KA1 | 0.005012 | 0.003128 | 280162.5 | 1198716 |
| 136 | RPS6KA2 | 0.004819 | 0.001997 | 56316.91 | 3836137 |
| 137 | RPS6KA3 | 0.004333 | 0.00528 | 1218337 | 8842769 |
| 138 | RRAS | 0.003674 | 0.002863 | 89885.17 | 271507.2 |
| 139 | BDNF | 0.005943 | 0.00339 | 115.5037 | 2512.222 |
| 140 | MAPK12 | 0.004837 | 0.003062 | 3712.622 | 529069.9 |
| 141 | MAP2K4 | 0.004743 | 0.005759 | 110081.6 | 246350.9 |
| 142 | SOS1 | 0.004565 | 0.004179 | 177000.4 | 667401 |
| 143 | SOS2 | 0.003757 | 0.005126 | 79581.07 | 239681.1 |
| 144 | SRF | 0.005498 | 0.005871 | 994707.4 | 622611.2 |
| 145 | BRAF | 0.004811 | 0.005045 | 3856.181 | 13824.99 |
| 146 | MAP3K7 | 0.004215 | 0.00432 | 34378.18 | 179435.3 |
| 147 | TGFB1 | 0.004745 | 0.006598 | 234958.5 | 9310816 |
| 148 | TGFB2 | 0.005722 | 0.007534 | 17833.16 | 111273.1 |
| 149 | TGFB3 | 0.002975 | 0.004345 | 148147.1 | 634219.6 |
| 150 | TGFBR1 | 0.003088 | 0.005213 | 122831.2 | 2580150 |
| 151 | TGFBR2 | 0.004491 | 0.006102 | 2702024 | 10105278 |
| 152 | TNF | 0.008044 | 0.002314 | 547.1939 | 252.9159 |
| 153 | TNFRSF1A | 0.002965 | 0.002167 | 7506121 | 4211749 |
| 154 | TRAF2 | 0.00473 | 0.007054 | 23299.23 | 448654.9 |
| 155 | CACNA1A | 0.003777 | 0.003247 | 10.78571 | 152.5963 |
| 156 | CACNA1B | 0.000623 | 0.000608 | 0.122857 | 403.3061 |
| 157 | CACNA1C | 0.004471 | 0.00309 | 936.7527 | 39918.89 |
| 158 | CACNA1D | 0.004012 | 0.006657 | 1606.826 | 56465.67 |
| 159 | CACNA1E | 0.000665 | 0.001153 | 124.1637 | 61065.18 |
| 160 | CACNA1F | 0.002506 | 0.00176 | 1.216735 | 271.2984 |
| 161 | MAP3K12 | 0.003772 | 0.006432 | 1968.08 | 8092.915 |
| 162 | CACNA1S | 0.000623 | 0.001573 | 0.132653 | 2460.858 |
| 163 | CACNA2D1 | 0.004428 | 0.002427 | 55.47959 | 608.1241 |
| 164 | CACNB1 | 0.003907 | 0.003289 | 240.7596 | 1556.99 |
| 165 | CACNB2 | 0.00329 | 0.002163 | 879.1939 | 23565 |
| 166 | CACNB3 | 0.003766 | 0.003467 | 749.8878 | 5815.021 |
| 167 | CACNB4 | 0.004333 | 0.003534 | 9.479592 | 4749.871 |
| 168 | IL1R2 | 0.004656 | 0.002318 | 4816.02 | 59579.51 |
| 169 | CACNG1 | 0.001256 | 0.001348 | 0.163673 | 149.1935 |
| 170 | MAPKAPK3 | 0.004976 | 0.006575 | 92360.48 | 1068318 |
| 171 | FGF23 | 0.001763 | 0.003178 | 3819.996 | 420.3494 |
| 172 | DUSP16 | 0.003696 | 0.005422 | 4837630 | 7432864 |
| 173 | CASP3 | 0.00363 | 0.004201 | 53428.78 | 371187.5 |
| 174 | PTPN5 | 0.002473 | 0.004478 | 1.012653 | 36.46082 |
| 175 | MAP4K3 | 0.003779 | 0.003952 | 61276.34 | 340702.7 |
| 176 | IKBKG | 0.005311 | 0.000721 | 182570.3 | 3001805 |
| 177 | MAPKAPK5 | 0.003949 | 0.005967 | 22376.04 | 156279.8 |
| 178 | MKNK1 | 0.001968 | 0.00678 | 44985.51 | 121428.2 |
| 179 | LAMTOR3 | 0.003707 | 0.007031 | 67675.17 | 207587.9 |
| 180 | FGF18 | 0.006022 | 0.00188 | 10.00367 | 103.3506 |
| 181 | FGF17 | 0.001948 | 0.001791 | 9.936327 | 118.2041 |
| 182 | FGF16 | 0.000623 | 0.001787 | 0 | 0.02 |
| 183 | CACNA1I | 0.003909 | 0.002779 | 84.5751 | 103772.7 |
| 184 | CACNA1H | 0.002799 | 0.001658 | 390012.1 | 3748438 |
| 185 | CACNA1G | 0.004024 | 0.001586 | 0.785306 | 16.52082 |
| 186 | RPS6KA4 | 0.004239 | 0.006418 | 50326.22 | 290317.4 |
| 187 | MAP3K14 | 0.004738 | 0.002024 | 512688.2 | 783038.4 |
| 188 | MAP3K6 | 0.00511 | 0.005078 | 10613.06 | 40564.84 |
| 189 | MAP3K13 | 0.004568 | 0.004584 | 10415.44 | 88410.08 |
| 190 | RPS6KA5 | 0.004978 | 0.006922 | 2163.291 | 3032.671 |
| 191 | CACNA2D2 | 0.005186 | 0.001525 | 86.31061 | 3190.082 |
| 192 | MAPKAPK2 | 0.006211 | 0.003922 | 948801.1 | 6050495 |
| 193 | CD14 | 0.003763 | 0.001202 | 1.29E+08 | 2.37E+08 |
| 194 | CACNA2D4 | 0.004483 | 0.001379 | 894.5241 | 3226.145 |
| 195 | MAP4K4 | 0.003929 | 0.004465 | 766608 | 26850257 |
| 196 | MAPK8IP1 | 0.003248 | 0.004554 | 12647.36 | 81879.77 |
| 197 | RAPGEF2 | 0.003642 | 0.005005 | 594912.4 | 971172.9 |
| 198 | FGF19 | 0.00265 | 0.003121 | 38967.29 | 539379.9 |
| 199 | CDC42 | 0.003569 | 0.004809 | 2657589 | 7614439 |
| 200 | MAP3K4 | 0.003791 | 0.004749 | 37267.84 | 173296.5 |
| 201 | NFKB1 | 0.004497 | 0.006757 | 490639.6 | 751180.5 |
| 202 | NFKB2 | 0.004723 | 0.004027 | 589838.2 | 2335128 |
| 203 | RELA | 0.003489 | 0.005177 | 520228.8 | 1500823 |
| 204 | RELB | 0.004682 | 0.006695 | 169102.1 | 603286.8 |
| 205 | ATF2 | 0.002693 | 0.004671 | 36123.52 | 189727.4 |
| 206 | DDIT3 | 0.005335 | 0.003135 | 111110 | 1745040 |
| 207 | MAX | 0.003534 | 0.0077 | 154132.9 | 211375.2 |
| 208 | MEF2C | 0.002795 | 0.005885 | 37739.75 | 212648.5 |
| 209 | TP53 | 0.003073 | 0.003429 | 147426.7 | 1104844 |
| 210 | CDC25B | 0.003356 | 0.00437 | 176637.4 | 2897750 |
| 211 | EGFR | 0.003857 | 0.005241 | 1150150 | 5154968 |
| 212 | FOS | 0.002587 | 0.003289 | 3.37E+08 | 86133193 |
| 213 | FGFR1 | 0.006943 | 0.0027 | 105500.3 | 348115.9 |
| 214 | FGFR3 | 0.005108 | 0.002831 | 346125 | 8934143 |
| 215 | FGFR2 | 0.004342 | 0.004007 | 2314236 | 13820396 |
| 216 | FGFR4 | 0.002912 | 0.004611 | 1479723 | 57925413 |
| 217 | TRAF6 | 0.003443 | 0.00654 | 5172.565 | 10280.55 |
| 218 | ATF4 | 0.005929 | 0.004095 | 12049563 | 45130029 |
| 219 | NTRK1 | 0.004901 | 0.003115 | 4.466939 | 22.90449 |
| 220 | NTRK2 | 0.005811 | 0.001624 | 7719.432 | 105778.8 |
| 221 | PDGFRA | 0.003334 | 0.003341 | 989994.7 | 1697151 |
| 222 | PDGFRB | 0.004383 | 0.003638 | 498072.6 | 12565258 |
| 223 | MAP3K2 | 0.003301 | 0.005237 | 120457.6 | 168698.4 |
| 224 | MAP3K3 | 0.002976 | 0.006865 | 90596.45 | 218745.7 |
| 225 | NFATC1 | 0.006252 | 0.003345 | 32921.76 | 49903.07 |
| 226 | NFATC3 | 0.003985 | 0.005603 | 178571.9 | 340480.2 |
| 227 | PLA2G4B | 0.002167 | 0.000876 | 0.612245 | 0.303673 |
| 228 | PLA2G4E | 0.002193 | 0.001352 | 1.108571 | 103.3882 |
| 229 | PLA2G4F | 0.001532 | 0.000754 | 0.53102 | 0.489796 |
| 230 | PLA2G4D | 0.004855 | 0.005244 | 112502.5 | 6967853 |
| 231 | STMN1 | 0.004031 | 0.001592 | 482.0637 | 229668.7 |
| 232 | MAPT | 0.005742 | 0.006316 | 9973273 | 6574528 |
| 233 | MYC | 0.003718 | 0.003983 | 1182.122 | 74403.07 |
| 234 | PLA2G4A | 0.00488 | 0.001549 | 59004.3 | 565116.9 |
| 235 | PLA2G4C | 0.005055 | 0.00172 | 13777.97 | 34007.89 |
| 236 | JMJD7-PLA2G4B | 0.003403 | 0.00401 | 30898449 | 8001024 |
| 237 | NR4A1 | 0.004081 | 0.008204 | 233961.9 | 327201.9 |
| 238 | CRK | 0.004194 | 0.004637 | 279379.1 | 2251791 |
| 239 | CRKL | 0.006242 | 0.002415 | 72022634 | 34563388 |
| 240 | JUN | 0.003486 | 0.003221 | 29055426 | 48853404 |
| 241 | JUND | 0.00423 | 0.005368 | 5913620 | 57312986 |
| 242 | FLNA | 0.003857 | 0.007566 | 5821307 | 34388216 |
| 243 | FLNB | 0.00374 | 0.001858 | 2959.756 | 16465394 |
| 244 | FLNC | 0.005304 | 0.001201 | 46194.96 | 501394.8 |
| 245 | ARRB2 | 0.00471 | 0.003251 | 309294.4 | 1438986 |
| 246 | NLK | 0.006502 | 0.004866 | 29026.94 | 146166.8 |
| 247 | MAP4K2 | 0.004548 | 0.004738 | 10623.41 | 90112.56 |
| 248 | HSPB1 | 0.00587 | 0.00364 | 7584076 | 5.14E+08 |
| 249 | STK3 | 0.005943 | 0.005647 | 17849.75 | 259804.1 |
| 250 | STK4 | 0.00339 | 0.005764 | 135102.3 | 392517.7 |

**Supplementary Table S1.** The PageRank scores of genes in “MAPK signaling pathway” for normal and cancer. We consolidate the information from **Section 1.1** – **Section 1.2** into one table with four columns, namely: (1) Gene index (1-250) (2) Gene symbol (3) The PageRank score of each gene in this pathway for normal (4) The PageRank score of each gene in this pathway for cancer. (5) The variance of gene expression for each gene across normal samples. (6) The variance of gene expression for each gene across cancer samples.
