# Supplementary material for: Pathways of topological rank analysis (PoTRA): a novel method to detect pathways involved in hepatocellular carcinoma
Source: PeerJ. 2018 Apr 9;6:e4571. doi: 10.7717/peerj.4571 (PMC5896492; doi:10.7717/peerj.4571)
Supplement: Table S2 — FDR adjusted P-values are below 0.05. E.comb.normal represents the number of the combined network for normal samples, while E.comb.case represents that for cancer samples. [file peerj-06-4571-s002.docx]

|  |  | Gene Counts | E.comb.normal | E.comb.case | Adjusted P value |
| --- | --- | --- | --- | --- | --- |
| 1 | Cytokine-cytokine receptor interaction | 253 | 125 | 85 | 0 |
| 2 | cAMP signaling pathway | 196 | 326 | 149 | 1.92713E-06 |
| 3 | Platinum drug resistance | 41 | 62 | 11 | 3.28115E-05 |
| 4 | alpha-Linolenic acid metabolism | 25 | 4 | 0 | 0.000108687 |
| 5 | Ascorbate and aldarate metabolism | 21 | 2 | 0 | 0.000108687 |
| 6 | Axon guidance | 167 | 308 | 130 | 0.000108687 |
| 7 | Neuroactive ligand-receptor interaction | 28 | 5 | 4 | 0.000108687 |
| 8 | p53 signaling pathway | 68 | 56 | 20 | 0.000108687 |
| 9 | Allograft rejection | 28 | 4 | 3 | 0.000109143 |
| 10 | Calcium signaling pathway | 179 | 293 | 97 | 0.000109143 |
| 11 | MAPK signaling pathway | 252 | 451 | 178 | 0.000109143 |
| 12 | Taste transduction | 29 | 8 | 3 | 0.000165454 |
| 13 | Phototransduction | 27 | 6 | 1 | 0.000240729 |
| 14 | Tuberculosis | 173 | 324 | 108 | 0.000244773 |
| 15 | Central carbon metabolism in cancer | 63 | 87 | 30 | 0.000729144 |
| 16 | cGMP-PKG signaling pathway | 158 | 206 | 107 | 0.000729144 |
| 17 | Maturity onset diabetes of the young | 24 | 6 | 5 | 0.000729144 |
| 18 | Ovarian steroidogenesis | 39 | 43 | 8 | 0.00076857 |
| 19 | Pathways in cancer | 310 | 694 | 318 | 0.00076857 |
| 20 | Adrenergic signaling in cardiomyocytes | 149 | 312 | 100 | 0.000946545 |
| 21 | Influenza A | 107 | 144 | 62 | 0.000946545 |
| 22 | Inflammatory mediator regulation of TRP channels | 91 | 105 | 32 | 0.001081704 |
| 23 | PI3K-Akt signaling pathway | 340 | 1536 | 702 | 0.001527379 |
| 24 | Ras signaling pathway | 226 | 638 | 255 | 0.001659245 |
| 25 | Epstein-Barr virus infection | 85 | 133 | 48 | 0.001665541 |
| 26 | Oxytocin signaling pathway | 157 | 251 | 110 | 0.001825885 |
| 27 | Hepatitis B | 134 | 210 | 98 | 0.001956781 |
| 28 | Aldosterone-regulated sodium reabsorption | 32 | 15 | 10 | 0.002224432 |
| 29 | Dilated cardiomyopathy | 76 | 181 | 98 | 0.002224432 |
| 30 | Alcoholism | 167 | 481 | 282 | 0.00225038 |
| 31 | HIF-1 signaling pathway | 102 | 173 | 71 | 0.002444877 |
| 32 | Tight junction | 125 | 221 | 84 | 0.002444877 |
| 33 | Drug metabolism - cytochrome P450 | 70 | 81 | 30 | 0.002612735 |
| 34 | Toxoplasmosis | 93 | 111 | 43 | 0.003416357 |
| 35 | Salivary secretion | 48 | 41 | 20 | 0.003658958 |
| 36 | Insulin secretion | 54 | 54 | 30 | 0.004205722 |
| 37 | Hepatitis C | 97 | 112 | 56 | 0.00445475 |
| 38 | Progesterone-mediated oocyte maturation | 89 | 90 | 45 | 0.004494478 |
| 39 | Natural killer cell mediated cytotoxicity | 134 | 205 | 114 | 0.006169437 |
| 40 | Type II diabetes mellitus | 47 | 43 | 10 | 0.006169437 |
| 41 | Toll-like receptor signaling pathway | 104 | 146 | 62 | 0.00645407 |
| 42 | Proteoglycans in cancer | 204 | 347 | 159 | 0.006590745 |
| 43 | Serotonergic synapse | 78 | 111 | 58 | 0.007023922 |
| 44 | Endocytosis | 109 | 351 | 132 | 0.009001174 |
| 45 | Neurotrophin signaling pathway | 117 | 255 | 102 | 0.009001174 |
| 46 | Breast cancer | 143 | 253 | 109 | 0.009052529 |
| 47 | Amyotrophic lateral sclerosis (ALS) | 36 | 21 | 6 | 0.009315863 |
| 48 | Epithelial cell signaling in Helicobacter pylori infection | 37 | 36 | 11 | 0.010838328 |
| 49 | Vascular smooth muscle contraction | 114 | 171 | 92 | 0.010838328 |
| 50 | Cocaine addiction | 42 | 37 | 16 | 0.011036994 |
| 51 | Measles | 102 | 141 | 74 | 0.013947996 |
| 52 | Gap junction | 88 | 116 | 55 | 0.014854222 |
| 53 | AMPK signaling pathway | 97 | 197 | 79 | 0.015099019 |
| 54 | Glutathione metabolism | 51 | 144 | 32 | 0.01528233 |
| 55 | B cell receptor signaling pathway | 70 | 106 | 39 | 0.016401527 |
| 56 | Apoptosis | 133 | 249 | 89 | 0.016440817 |
| 57 | Glycosphingolipid biosynthesis - globo and isoglobo series | 14 | 19 | 2 | 0.01827847 |
| 58 | Selenocompound metabolism | 14 | 16 | 2 | 0.01827847 |
| 59 | Alzheimer's disease | 48 | 43 | 17 | 0.020786715 |
| 60 | Endocrine resistance | 95 | 189 | 91 | 0.020786715 |
| 61 | Glioma | 66 | 134 | 47 | 0.020786715 |
| 62 | Insulin signaling pathway | 139 | 251 | 100 | 0.020786715 |
| 63 | NOD-like receptor signaling pathway | 48 | 89 | 41 | 0.020786715 |
| 64 | Chagas disease (American trypanosomiasis) | 89 | 127 | 65 | 0.022105426 |
| 65 | Cell adhesion molecules (CAMs) | 94 | 67 | 48 | 0.023256813 |
| 66 | Amoebiasis | 44 | 37 | 18 | 0.023611696 |
| 67 | Amphetamine addiction | 62 | 98 | 38 | 0.023611696 |
| 68 | Small cell lung cancer | 83 | 194 | 77 | 0.023611696 |
| 69 | Folate biosynthesis | 14 | 9 | 1 | 0.024042451 |
| 70 | Adipocytokine signaling pathway | 63 | 86 | 28 | 0.025057971 |
| 71 | Insulin resistance | 94 | 139 | 45 | 0.027377045 |
| 72 | TNF signaling pathway | 72 | 100 | 44 | 0.029020879 |
| 73 | Herpes simplex infection | 104 | 146 | 74 | 0.02912229 |
| 74 | Glutamatergic synapse | 89 | 160 | 62 | 0.029860673 |
| 75 | Oocyte meiosis | 120 | 349 | 253 | 0.031453635 |
| 76 | VEGF signaling pathway | 61 | 105 | 38 | 0.032143976 |
| 77 | Non-small cell lung cancer | 54 | 93 | 40 | 0.032875602 |
| 78 | Wnt signaling pathway | 137 | 351 | 160 | 0.032875602 |
| 79 | Porphyrin and chlorophyll metabolism | 39 | 22 | 9 | 0.034284952 |
| 80 | Hippo signaling pathway | 151 | 384 | 179 | 0.034762983 |
| 81 | Histidine metabolism | 23 | 11 | 4 | 0.034762983 |
| 82 | Leishmaniasis | 50 | 70 | 28 | 0.034762983 |
| 83 | Rap1 signaling pathway | 208 | 589 | 277 | 0.034762983 |
| 84 | Regulation of actin cytoskeleton | 186 | 485 | 226 | 0.034762983 |
| 85 | Glycerophospholipid metabolism | 94 | 448 | 83 | 0.03500119 |
| 86 | Linoleic acid metabolism | 29 | 23 | 7 | 0.036791435 |
| 87 | Osteoclast differentiation | 123 | 299 | 202 | 0.037104666 |
| 88 | mTOR signaling pathway | 144 | 289 | 119 | 0.037618347 |
| 89 | Bile secretion | 29 | 23 | 7 | 0.041999322 |
| 90 | Parkinson's disease | 29 | 18 | 7 | 0.041999322 |
| 91 | HTLV-I infection | 194 | 379 | 229 | 0.042040499 |
| 92 | EGFR tyrosine kinase inhibitor resistance | 81 | 178 | 65 | 0.042231193 |
| 93 | Cardiac muscle contraction | 13 | 4 | 2 | 0.042421633 |
| 94 | GABAergic synapse | 66 | 154 | 64 | 0.042436753 |
| 95 | Hedgehog signaling pathway | 47 | 103 | 56 | 0.042436753 |
| 96 | Legionellosis | 40 | 23 | 10 | 0.042436753 |
| 97 | Fc gamma R-mediated phagocytosis | 93 | 225 | 109 | 0.043768317 |
| 98 | Hypertrophic cardiomyopathy (HCM) | 25 | 12 | 9 | 0.043768317 |
| 99 | Melanogenesis | 101 | 158 | 82 | 0.045457628 |
| 100 | Focal adhesion | 203 | 1259 | 573 | 0.046594555 |

**Supplementary Table S2. The significant KEGG pathways identified by PoTRA for HCC using the Kolmogorov–Smirnov test under the constructed network combining the correlation network and the pre-defined KEGG network.** FDR adjusted P-values are below 0.05. E.comb.normal represents the number of the combined network for normal samples, while E.comb.case represents that for cancer samples.
