# Supplementary material for: Pathways of topological rank analysis (PoTRA): a novel method to detect pathways involved in hepatocellular carcinoma
Source: PeerJ. 2018 Apr 9;6:e4571. doi: 10.7717/peerj.4571 (PMC5896492; doi:10.7717/peerj.4571)
Supplement: Table S3 — FDR adjusted P-values are below 0.05. E.comb.normal represents the number of the combined network for normal samples, while E.comb.case represents that for cancer samples. [file peerj-06-4571-s003.docx]

|  |  | Gene Counts | E.comb.normal | E.comb.case | Adjusted P value |
| --- | --- | --- | --- | --- | --- |
| 1 | Cytokine-cytokine receptor interaction | 253 | 106 | 68 | 0 |
| 2 | MAPK signaling pathway | 252 | 413 | 136 | 4.932E-09 |
| 3 | Calcium signaling pathway | 179 | 221 | 84 | 6.73355E-09 |
| 4 | cAMP signaling pathway | 196 | 277 | 105 | 1.23848E-08 |
| 5 | cGMP-PKG signaling pathway | 158 | 173 | 70 | 2.192E-07 |
| 6 | Adrenergic signaling in cardiomyocytes | 149 | 246 | 61 | 1.02946E-06 |
| 7 | HIF-1 signaling pathway | 102 | 159 | 52 | 2.11323E-06 |
| 8 | Toll-like receptor signaling pathway | 104 | 133 | 36 | 4.96244E-06 |
| 9 | Insulin resistance | 94 | 132 | 35 | 1.16724E-05 |
| 10 | alpha-Linolenic acid metabolism | 25 | 3 | 2 | 1.34032E-05 |
| 11 | Proteoglycans in cancer | 204 | 316 | 119 | 1.34032E-05 |
| 12 | Epstein-Barr virus infection | 85 | 131 | 43 | 1.52461E-05 |
| 13 | Neuroactive ligand-receptor interaction | 28 | 4 | 0 | 1.52461E-05 |
| 14 | Allograft rejection | 28 | 3 | 3 | 1.95453E-05 |
| 15 | Alcoholism | 167 | 417 | 329 | 2.08925E-05 |
| 16 | Non-alcoholic fatty liver disease (NAFLD) | 73 | 84 | 23 | 0.000022194 |
| 17 | p53 signaling pathway | 68 | 57 | 20 | 0.000022194 |
| 18 | Pathways in cancer | 310 | 648 | 299 | 0.000022194 |
| 19 | Tuberculosis | 173 | 287 | 102 | 0.000022194 |
| 20 | Drug metabolism - other enzymes | 44 | 42 | 9 | 2.88352E-05 |
| 21 | FoxO signaling pathway | 126 | 159 | 65 | 2.88945E-05 |
| 22 | Amino sugar and nucleotide sugar metabolism | 43 | 59 | 16 | 0.00003562 |
| 23 | Phototransduction | 27 | 5 | 1 | 0.00003562 |
| 24 | Ras signaling pathway | 226 | 556 | 217 | 4.59224E-05 |
| 25 | Progesterone-mediated oocyte maturation | 89 | 89 | 31 | 4.94254E-05 |
| 26 | Influenza A | 107 | 132 | 53 | 5.63222E-05 |
| 27 | Type II diabetes mellitus | 47 | 49 | 8 | 0.00009316 |
| 28 | Apoptosis | 133 | 231 | 87 | 0.000102986 |
| 29 | Maturity onset diabetes of the young | 24 | 4 | 4 | 0.000107773 |
| 30 | Osteoclast differentiation | 123 | 286 | 146 | 0.000128161 |
| 31 | Cell adhesion molecules (CAMs) | 94 | 58 | 34 | 0.000196081 |
| 32 | NOD-like receptor signaling pathway | 48 | 89 | 28 | 0.000266747 |
| 33 | PI3K-Akt signaling pathway | 340 | 1279 | 573 | 0.000266747 |
| 34 | Insulin secretion | 54 | 50 | 17 | 0.000364811 |
| 35 | Ovarian steroidogenesis | 39 | 39 | 14 | 0.000407956 |
| 36 | Dilated cardiomyopathy | 76 | 134 | 77 | 0.000427292 |
| 37 | Oxytocin signaling pathway | 157 | 221 | 107 | 0.000474453 |
| 38 | Salivary secretion | 48 | 35 | 11 | 0.000586641 |
| 39 | Measles | 102 | 134 | 60 | 0.000598005 |
| 40 | Platinum drug resistance | 41 | 61 | 15 | 0.000774424 |
| 41 | Chemical carcinogenesis | 68 | 123 | 21 | 0.000849967 |
| 42 | Vascular smooth muscle contraction | 114 | 149 | 69 | 0.000849967 |
| 43 | Tight junction | 125 | 208 | 78 | 0.001031965 |
| 44 | Insulin signaling pathway | 139 | 237 | 66 | 0.001080793 |
| 45 | Rap1 signaling pathway | 208 | 466 | 220 | 0.001080793 |
| 46 | Glucagon signaling pathway | 86 | 184 | 68 | 0.001141455 |
| 47 | Hepatitis B | 134 | 196 | 93 | 0.001141455 |
| 48 | Central carbon metabolism in cancer | 63 | 83 | 21 | 0.001196441 |
| 49 | Breast cancer | 143 | 213 | 102 | 0.001270067 |
| 50 | Amyotrophic lateral sclerosis (ALS) | 36 | 13 | 10 | 0.001326053 |
| 51 | Chagas disease (American trypanosomiasis) | 89 | 121 | 48 | 0.001544574 |
| 52 | Taste transduction | 29 | 5 | 11 | 0.001544574 |
| 53 | Serotonergic synapse | 78 | 83 | 52 | 0.00156178 |
| 54 | Neurotrophin signaling pathway | 117 | 240 | 91 | 0.00157548 |
| 55 | GABAergic synapse | 66 | 122 | 70 | 0.001583864 |
| 56 | Inflammatory mediator regulation of TRP channels | 91 | 92 | 33 | 0.001583864 |
| 57 | Regulation of lipolysis in adipocytes | 51 | 63 | 21 | 0.001659519 |
| 58 | Histidine metabolism | 23 | 6 | 2 | 0.001849203 |
| 59 | Glycerolipid metabolism | 57 | 109 | 26 | 0.001874717 |
| 60 | Alzheimer's disease | 48 | 35 | 11 | 0.002138627 |
| 61 | Melanogenesis | 101 | 130 | 64 | 0.002138627 |
| 62 | Pyruvate metabolism | 39 | 75 | 14 | 0.002145137 |
| 63 | Axon guidance | 167 | 279 | 133 | 0.002209254 |
| 64 | Endocrine and other factor-regulated calcium reabsorption | 35 | 15 | 3 | 0.002209254 |
| 65 | Legionellosis | 40 | 19 | 9 | 0.002520294 |
| 66 | Hepatitis C | 97 | 110 | 43 | 0.002526574 |
| 67 | NF-kappa B signaling pathway | 81 | 149 | 50 | 0.002953913 |
| 68 | HTLV-I infection | 194 | 353 | 194 | 0.003125887 |
| 69 | B cell receptor signaling pathway | 70 | 117 | 33 | 0.00356334 |
| 70 | Endocrine resistance | 95 | 188 | 72 | 0.00356334 |
| 71 | Gap junction | 88 | 109 | 41 | 0.00356334 |
| 72 | AMPK signaling pathway | 97 | 185 | 62 | 0.003780367 |
| 73 | Ascorbate and aldarate metabolism | 21 | 1 | 5 | 0.003784895 |
| 74 | Glycine, serine and threonine metabolism | 35 | 52 | 10 | 0.003924075 |
| 75 | Toxoplasmosis | 93 | 105 | 35 | 0.005025275 |
| 76 | Endocytosis | 109 | 320 | 125 | 0.005283883 |
| 77 | Natural killer cell mediated cytotoxicity | 134 | 219 | 92 | 0.005283883 |
| 78 | Aldosterone-regulated sodium reabsorption | 32 | 19 | 6 | 0.006054212 |
| 79 | Regulation of actin cytoskeleton | 186 | 464 | 203 | 0.006054212 |
| 80 | Herpes simplex infection | 104 | 139 | 55 | 0.006110241 |
| 81 | Oocyte meiosis | 120 | 320 | 183 | 0.007314948 |
| 82 | Bile secretion | 29 | 21 | 5 | 0.007460953 |
| 83 | Fatty acid elongation | 25 | 25 | 6 | 0.007460953 |
| 84 | Hypertrophic cardiomyopathy (HCM) | 25 | 10 | 10 | 0.007460953 |
| 85 | Inflammatory bowel disease (IBD) | 48 | 37 | 12 | 0.007460953 |
| 86 | Glioma | 66 | 138 | 41 | 0.00789559 |
| 87 | Arginine and proline metabolism | 50 | 46 | 11 | 0.008368284 |
| 88 | Glutamatergic synapse | 89 | 139 | 76 | 0.008845773 |
| 89 | Glyoxylate and dicarboxylate metabolism | 26 | 31 | 7 | 0.009154273 |
| 90 | Estrogen signaling pathway | 89 | 123 | 49 | 0.009210388 |
| 91 | Protein processing in endoplasmic reticulum | 51 | 66 | 27 | 0.009210388 |
| 92 | Acute myeloid leukemia | 57 | 96 | 35 | 0.010465642 |
| 93 | mTOR signaling pathway | 144 | 280 | 104 | 0.010772855 |
| 94 | Aminoacyl-tRNA biosynthesis | 13 | 14 | 3 | 0.011351892 |
| 95 | Fructose and mannose metabolism | 32 | 54 | 15 | 0.012486928 |
| 96 | Cocaine addiction | 42 | 30 | 12 | 0.012731529 |
| 97 | Porphyrin and chlorophyll metabolism | 39 | 26 | 8 | 0.012781286 |
| 98 | Linoleic acid metabolism | 29 | 21 | 9 | 0.013239389 |
| 99 | African trypanosomiasis | 24 | 10 | 1 | 0.013583561 |
| 100 | Choline metabolism in cancer | 82 | 140 | 46 | 0.013785282 |
| 101 | RNA transport | 133 | 213 | 128 | 0.014149282 |
| 102 | Colorectal cancer | 49 | 67 | 33 | 0.014464255 |
| 103 | Leishmaniasis | 50 | 69 | 21 | 0.014464255 |
| 104 | Dopaminergic synapse | 124 | 280 | 150 | 0.01448394 |
| 105 | Sphingolipid signaling pathway | 98 | 197 | 64 | 0.01448394 |
| 106 | Amoebiasis | 44 | 39 | 12 | 0.01508525 |
| 107 | Amphetamine addiction | 62 | 86 | 42 | 0.01508525 |
| 108 | Glutathione metabolism | 51 | 124 | 15 | 0.015528588 |
| 109 | Tyrosine metabolism | 34 | 50 | 16 | 0.015624564 |
| 110 | Adipocytokine signaling pathway | 63 | 79 | 18 | 0.016028072 |
| 111 | Oxidative phosphorylation | 47 | 78 | 42 | 0.016554248 |
| 112 | Complement and coagulation cascades | 52 | 39 | 13 | 0.016776448 |
| 113 | Pathogenic Escherichia coli infection | 40 | 38 | 24 | 0.017336812 |
| 114 | Signaling pathways regulating pluripotency of stem cells | 112 | 180 | 78 | 0.017815754 |
| 115 | Thyroid hormone signaling pathway | 112 | 306 | 116 | 0.017815754 |
| 116 | Chronic myeloid leukemia | 73 | 156 | 70 | 0.019538844 |
| 117 | Wnt signaling pathway | 137 | 316 | 138 | 0.021824903 |
| 118 | Metabolism of xenobiotics by cytochrome P450 | 72 | 192 | 48 | 0.022594695 |
| 119 | Small cell lung cancer | 83 | 186 | 103 | 0.022594695 |
| 120 | Lysine degradation | 55 | 111 | 40 | 0.023657837 |
| 121 | Retinol metabolism | 65 | 194 | 56 | 0.024148033 |
| 122 | Chemokine signaling pathway | 187 | 734 | 351 | 0.025410929 |
| 123 | Renin secretion | 50 | 48 | 19 | 0.02641016 |
| 124 | Prolactin signaling pathway | 72 | 112 | 46 | 0.029131722 |
| 125 | Starch and sucrose metabolism | 36 | 40 | 10 | 0.030179415 |
| 126 | Jak-STAT signaling pathway | 158 | 576 | 301 | 0.030199272 |
| 127 | Cardiac muscle contraction | 13 | 4 | 2 | 0.030911888 |
| 128 | Glycosphingolipid biosynthesis - globo and isoglobo series | 14 | 15 | 2 | 0.030911888 |
| 129 | Hippo signaling pathway | 151 | 350 | 190 | 0.032671256 |
| 130 | Primary bile acid biosynthesis | 17 | 13 | 4 | 0.035663464 |
| 131 | Melanoma | 69 | 112 | 38 | 0.03792368 |
| 132 | N-Glycan biosynthesis | 49 | 103 | 28 | 0.037946629 |
| 133 | Cysteine and methionine metabolism | 43 | 46 | 15 | 0.038456327 |
| 134 | Viral myocarditis | 26 | 11 | 3 | 0.038663994 |
| 135 | Longevity regulating pathway - multiple species | 63 | 134 | 38 | 0.041020544 |
| 136 | Bacterial invasion of epithelial cells | 57 | 94 | 47 | 0.044182429 |
| 137 | Gastric acid secretion | 57 | 73 | 29 | 0.044182429 |
| 138 | Propanoate metabolism | 32 | 67 | 19 | 0.044182429 |
| 139 | EGFR tyrosine kinase inhibitor resistance | 81 | 170 | 58 | 0.04540383 |
| 140 | AGE-RAGE signaling pathway in diabetic complications | 91 | 213 | 93 | 0.0479263 |

**Supplementary Table S3. The significant KEGG pathways identified by PoTRA for hepatitis C-induced HCC using the Kolmogorov–Smirnov test under the constructed network combining the correlation network and the pre-defined KEGG network.** FDR adjusted P-values are below 0.05. E.comb.normal represents the number of the combined network for normal samples, while E.comb.case represents that for cancer samples.
