# Supplementary material for: Pathways of topological rank analysis (PoTRA): a novel method to detect pathways involved in hepatocellular carcinoma
Source: PeerJ. 2018 Apr 9;6:e4571. doi: 10.7717/peerj.4571 (PMC5896492; doi:10.7717/peerj.4571)
Supplement: Table S4 — FDR adjusted P-values are below 0.05. E.comb.normal represents the number of the combined network for normal samples, while E.comb.case represents that for cancer samples. [file peerj-06-4571-s004.docx]

|  |  | Gene Counts | E.comb.normal | E.comb.case | Adjusted P value |
| --- | --- | --- | --- | --- | --- |
|  |  |  |  |  |  |
|  |  |  |  |  |  |
| 1 | Adrenergic signaling in cardiomyocytes | 149 | 86 | 21 | 0 |
| 2 | Breast cancer | 143 | 72 | 46 | 0 |
| 3 | Calcium signaling pathway | 179 | 63 | 23 | 0 |
| 4 | cAMP signaling pathway | 196 | 113 | 52 | 0 |
| 5 | cGMP-PKG signaling pathway | 158 | 74 | 36 | 0 |
| 6 | Cytokine-cytokine receptor interaction | 253 | 56 | 42 | 0 |
| 7 | MAPK signaling pathway | 252 | 228 | 64 | 0 |
| 8 | PI3K-Akt signaling pathway | 340 | 613 | 319 | 1.68967E-14 |
| 9 | Inflammatory mediator regulation of TRP channels | 91 | 30 | 8 | 3.58691E-14 |
| 10 | Tuberculosis | 173 | 160 | 43 | 3.58691E-14 |
| 11 | Oxytocin signaling pathway | 157 | 99 | 32 | 3.425E-13 |
| 12 | Pathways in cancer | 310 | 355 | 136 | 7.28057E-13 |
| 13 | Ras signaling pathway | 226 | 253 | 127 | 7.28057E-13 |
| 14 | Alcoholism | 167 | 242 | 137 | 9.22467E-13 |
| 15 | Signaling pathways regulating pluripotency of stem cells | 112 | 70 | 44 | 3.09963E-12 |
| 16 | Vascular smooth muscle contraction | 114 | 73 | 33 | 1.05087E-11 |
| 17 | Apoptosis | 133 | 148 | 22 | 1.73533E-11 |
| 18 | Rap1 signaling pathway | 208 | 190 | 134 | 1.06283E-10 |
| 19 | Insulin secretion | 54 | 17 | 7 | 2.6715E-10 |
| 20 | Serotonergic synapse | 78 | 29 | 25 | 3.014E-10 |
| 21 | Taste transduction | 29 | 0 | 1 | 3.014E-10 |
| 22 | Natural killer cell mediated cytotoxicity | 134 | 101 | 49 | 7.36226E-10 |
| 23 | Glutamatergic synapse | 89 | 49 | 25 | 8.23142E-10 |
| 24 | Ovarian steroidogenesis | 39 | 9 | 4 | 1.13984E-09 |
| 25 | Cell adhesion molecules (CAMs) | 94 | 39 | 31 | 1.29623E-09 |
| 26 | Axon guidance | 167 | 132 | 51 | 1.77593E-09 |
| 27 | Salivary secretion | 48 | 12 | 2 | 2.74979E-09 |
| 28 | Proteoglycans in cancer | 204 | 180 | 59 | 3.77931E-09 |
| 29 | Amyotrophic lateral sclerosis (ALS) | 36 | 5 | 1 | 6.36593E-09 |
| 30 | Insulin resistance | 94 | 53 | 21 | 7.7869E-09 |
| 31 | Toll-like receptor signaling pathway | 104 | 87 | 16 | 8.30563E-09 |
| 32 | Neuroactive ligand-receptor interaction | 28 | 2 | 1 | 9.0503E-09 |
| 33 | HTLV-I infection | 194 | 215 | 108 | 1.08794E-08 |
| 34 | Endocrine and other factor-regulated calcium reabsorption | 35 | 5 | 3 | 1.40914E-08 |
| 35 | Cholinergic synapse | 94 | 89 | 35 | 1.72772E-08 |
| 36 | EGFR tyrosine kinase inhibitor resistance | 81 | 86 | 17 | 3.91005E-08 |
| 37 | Aldosterone-regulated sodium reabsorption | 32 | 4 | 4 | 4.5621E-08 |
| 38 | FoxO signaling pathway | 126 | 85 | 36 | 4.5621E-08 |
| 39 | Hepatitis B | 134 | 135 | 47 | 4.5621E-08 |
| 40 | Dopaminergic synapse | 124 | 155 | 45 | 5.21268E-08 |
| 41 | Melanogenesis | 101 | 64 | 29 | 6.17805E-08 |
| 42 | Non-alcoholic fatty liver disease (NAFLD) | 73 | 53 | 8 | 9.11209E-08 |
| 43 | HIF-1 signaling pathway | 102 | 78 | 17 | 1.64983E-07 |
| 44 | Neurotrophin signaling pathway | 117 | 139 | 47 | 1.64983E-07 |
| 45 | Progesterone-mediated oocyte maturation | 89 | 46 | 10 | 1.64983E-07 |
| 46 | Toxoplasmosis | 93 | 68 | 20 | 1.64983E-07 |
| 47 | Sphingolipid signaling pathway | 98 | 115 | 30 | 1.70551E-07 |
| 48 | TNF signaling pathway | 72 | 65 | 13 | 1.70551E-07 |
| 49 | Drug metabolism - cytochrome P450 | 70 | 37 | 26 | 2.60827E-07 |
| 50 | GABAergic synapse | 66 | 43 | 25 | 2.60827E-07 |
| 51 | NOD-like receptor signaling pathway | 48 | 64 | 16 | 2.60827E-07 |
| 52 | Gap junction | 88 | 51 | 22 | 3.22079E-07 |
| 53 | Estrogen signaling pathway | 89 | 65 | 22 | 3.84107E-07 |
| 54 | Circadian entrainment | 96 | 92 | 40 | 5.67571E-07 |
| 55 | Hippo signaling pathway | 151 | 206 | 104 | 5.67571E-07 |
| 56 | Influenza A | 107 | 80 | 28 | 6.63368E-07 |
| 57 | Tight junction | 125 | 138 | 29 | 6.70828E-07 |
| 58 | Phototransduction | 27 | 2 | 4 | 7.47695E-07 |
| 59 | Adipocytokine signaling pathway | 63 | 44 | 1 | 7.86066E-07 |
| 60 | Central carbon metabolism in cancer | 63 | 51 | 6 | 7.86066E-07 |
| 61 | mTOR signaling pathway | 144 | 179 | 43 | 8.8829E-07 |
| 62 | Acute myeloid leukemia | 57 | 69 | 10 | 1.20908E-06 |
| 63 | Legionellosis | 40 | 9 | 1 | 1.39997E-06 |
| 64 | Alzheimer's disease | 48 | 20 | 2 | 2.1372E-06 |
| 65 | p53 signaling pathway | 68 | 33 | 14 | 2.39542E-06 |
| 66 | Measles | 102 | 100 | 29 | 2.73182E-06 |
| 67 | Arginine and proline metabolism | 50 | 32 | 1 | 2.74397E-06 |
| 68 | Hepatitis C | 97 | 79 | 29 | 2.74397E-06 |
| 69 | Gastric acid secretion | 57 | 23 | 11 | 3.06097E-06 |
| 70 | Herpes simplex infection | 104 | 109 | 26 | 3.43465E-06 |
| 71 | Insulin signaling pathway | 139 | 147 | 44 | 3.58483E-06 |
| 72 | Non-small cell lung cancer | 54 | 60 | 17 | 3.71589E-06 |
| 73 | Allograft rejection | 28 | 3 | 2 | 3.96189E-06 |
| 74 | Porphyrin and chlorophyll metabolism | 39 | 12 | 8 | 4.47053E-06 |
| 75 | Regulation of lipolysis in adipocytes | 51 | 24 | 3 | 4.47053E-06 |
| 76 | Oocyte meiosis | 120 | 224 | 135 | 6.35821E-06 |
| 77 | Small cell lung cancer | 83 | 138 | 38 | 6.35821E-06 |
| 78 | Osteoclast differentiation | 123 | 189 | 98 | 7.21418E-06 |
| 79 | African trypanosomiasis | 24 | 4 | 1 | 9.26864E-06 |
| 80 | Maturity onset diabetes of the young | 24 | 3 | 4 | 9.26864E-06 |
| 81 | Lysine degradation | 55 | 80 | 13 | 1.16951E-05 |
| 82 | B cell receptor signaling pathway | 70 | 74 | 18 | 1.39579E-05 |
| 83 | Melanoma | 69 | 50 | 21 | 1.39579E-05 |
| 84 | VEGF signaling pathway | 61 | 48 | 7 | 1.39579E-05 |
| 85 | Endometrial cancer | 45 | 45 | 11 | 1.7587E-05 |
| 86 | AGE-RAGE signaling pathway in diabetic complications | 91 | 111 | 26 | 1.91485E-05 |
| 87 | Amphetamine addiction | 62 | 48 | 19 | 2.0083E-05 |
| 88 | Regulation of actin cytoskeleton | 186 | 296 | 108 | 2.24319E-05 |
| 89 | Renin secretion | 50 | 18 | 5 | 2.24319E-05 |
| 90 | Shigellosis | 51 | 74 | 14 | 2.24319E-05 |
| 91 | Chagas disease (American trypanosomiasis) | 89 | 86 | 22 | 2.89189E-05 |
| 92 | Thyroid hormone signaling pathway | 112 | 185 | 80 | 2.89615E-05 |
| 93 | AMPK signaling pathway | 97 | 107 | 37 | 3.43957E-05 |
| 94 | Amoebiasis | 44 | 24 | 4 | 3.46105E-05 |
| 95 | Fc gamma R-mediated phagocytosis | 93 | 136 | 30 | 3.62479E-05 |
| 96 | ErbB signaling pathway | 88 | 82 | 29 | 4.37835E-05 |
| 97 | Oxidative phosphorylation | 47 | 68 | 4 | 4.38959E-05 |
| 98 | Hypertrophic cardiomyopathy (HCM) | 25 | 5 | 4 | 5.28626E-05 |
| 99 | Chronic myeloid leukemia | 73 | 100 | 43 | 0.000054252 |
| 100 | Type II diabetes mellitus | 47 | 21 | 5 | 7.02634E-05 |
| 101 | Salmonella infection | 72 | 102 | 27 | 8.59608E-05 |
| 102 | Glycerophospholipid metabolism | 94 | 141 | 34 | 8.77864E-05 |
| 103 | RNA transport | 133 | 188 | 79 | 9.35288E-05 |
| 104 | Platinum drug resistance | 41 | 38 | 6 | 0.000111166 |
| 105 | GnRH signaling pathway | 85 | 64 | 22 | 0.000115028 |
| 106 | Jak-STAT signaling pathway | 158 | 324 | 137 | 0.00012266 |
| 107 | Platelet activation | 118 | 130 | 46 | 0.000131419 |
| 108 | Cocaine addiction | 42 | 16 | 4 | 0.000138508 |
| 109 | Chemokine signaling pathway | 187 | 398 | 148 | 0.000150202 |
| 110 | Chemical carcinogenesis | 68 | 71 | 41 | 0.00015033 |
| 111 | Prostate cancer | 87 | 116 | 45 | 0.000157305 |
| 112 | Endocrine resistance | 95 | 109 | 46 | 0.000196165 |
| 113 | Inflammatory bowel disease (IBD) | 48 | 28 | 4 | 0.000200693 |
| 114 | Longevity regulating pathway | 84 | 109 | 28 | 0.00021372 |
| 115 | Bile secretion | 29 | 10 | 0 | 0.000220137 |
| 116 | Parkinson's disease | 29 | 9 | 1 | 0.000220137 |
| 117 | Primary bile acid biosynthesis | 17 | 14 | 1 | 0.000223612 |
| 118 | Glycosylphosphatidylinositol(GPI)-anchor biosynthesis | 23 | 34 | 3 | 0.000259837 |
| 119 | Choline metabolism in cancer | 82 | 73 | 20 | 0.000285798 |
| 120 | Aldosterone synthesis and secretion | 65 | 72 | 27 | 0.000302054 |
| 121 | Drug metabolism - other enzymes | 44 | 24 | 6 | 0.000328308 |
| 122 | Glioma | 66 | 72 | 22 | 0.00034102 |
| 123 | Phospholipase D signaling pathway | 116 | 123 | 50 | 0.00034102 |
| 124 | Epstein-Barr virus infection | 85 | 84 | 22 | 0.000346432 |
| 125 | Prolactin signaling pathway | 72 | 69 | 26 | 0.000363805 |
| 126 | Cytosolic DNA-sensing pathway | 21 | 23 | 3 | 0.000381559 |
| 127 | Glucagon signaling pathway | 86 | 105 | 42 | 0.000419241 |
| 128 | NF-kappa B signaling pathway | 81 | 101 | 26 | 0.000419241 |
| 129 | Valine, leucine and isoleucine degradation | 48 | 154 | 34 | 0.000431278 |
| 130 | Linoleic acid metabolism | 29 | 15 | 10 | 0.000447524 |
| 131 | Long-term depression | 59 | 41 | 8 | 0.000454089 |
| 132 | Pathogenic Escherichia coli infection | 40 | 31 | 6 | 0.000495932 |
| 133 | Wnt signaling pathway | 137 | 137 | 70 | 0.000500993 |
| 134 | Protein processing in endoplasmic reticulum | 51 | 52 | 13 | 0.000599749 |
| 135 | Thyroid hormone synthesis | 46 | 29 | 7 | 0.000674167 |
| 136 | Complement and coagulation cascades | 52 | 36 | 8 | 0.000692461 |
| 137 | Glyoxylate and dicarboxylate metabolism | 26 | 28 | 4 | 0.000692461 |
| 138 | SNARE interactions in vesicular transport | 26 | 53 | 8 | 0.000692461 |
| 139 | Long-term potentiation | 67 | 59 | 17 | 0.000722796 |
| 140 | Leukocyte transendothelial migration | 85 | 118 | 41 | 0.000732567 |
| 141 | Metabolism of xenobiotics by cytochrome P450 | 72 | 109 | 57 | 0.000808653 |
| 142 | Focal adhesion | 203 | 580 | 276 | 0.000931838 |
| 143 | beta-Alanine metabolism | 31 | 35 | 11 | 0.000986248 |
| 144 | Antigen processing and presentation | 62 | 43 | 46 | 0.001069465 |
| 145 | Leishmaniasis | 50 | 50 | 15 | 0.001069465 |
| 146 | Galactose metabolism | 28 | 14 | 7 | 0.001206692 |
| 147 | Fructose and mannose metabolism | 32 | 31 | 8 | 0.001242118 |
| 148 | Glycerolipid metabolism | 57 | 39 | 23 | 0.001605189 |
| 149 | Glycosphingolipid biosynthesis - lacto and neolacto series | 26 | 37 | 2 | 0.001918779 |
| 150 | Intestinal immune network for IgA production | 28 | 9 | 1 | 0.001918779 |
| 151 | Viral myocarditis | 26 | 7 | 0 | 0.001918779 |
| 152 | RIG-I-like receptor signaling pathway | 48 | 57 | 17 | 0.001940138 |
| 153 | Colorectal cancer | 49 | 53 | 16 | 0.002226738 |
| 154 | Malaria | 11 | 1 | 0 | 0.002226738 |
| 155 | Taurine and hypotaurine metabolism | 11 | 1 | 0 | 0.002226738 |
| 156 | Histidine metabolism | 23 | 6 | 0 | 0.002247816 |
| 157 | TGF-beta signaling pathway | 73 | 62 | 47 | 0.002471586 |
| 158 | Tryptophan metabolism | 40 | 34 | 5 | 0.002509607 |
| 159 | ECM-receptor interaction | 81 | 170 | 88 | 0.002531447 |
| 160 | Endocytosis | 109 | 180 | 66 | 0.002531447 |
| 161 | Steroid biosynthesis | 20 | 14 | 1 | 0.002531447 |
| 162 | T cell receptor signaling pathway | 95 | 110 | 46 | 0.002531447 |
| 163 | Pyrimidine metabolism | 105 | 555 | 131 | 0.002625387 |
| 164 | Renal cell carcinoma | 57 | 50 | 17 | 0.002625387 |
| 165 | Rheumatoid arthritis | 17 | 3 | 4 | 0.002676194 |
| 166 | Sphingolipid metabolism | 47 | 103 | 21 | 0.00332816 |
| 167 | Adherens junction | 71 | 96 | 37 | 0.003572141 |
| 168 | Fatty acid elongation | 25 | 21 | 1 | 0.00375892 |
| 169 | Notch signaling pathway | 48 | 52 | 58 | 0.003774365 |
| 170 | Synaptic vesicle cycle | 18 | 18 | 2 | 0.003857984 |
| 171 | Arachidonic acid metabolism | 62 | 40 | 20 | 0.004522926 |
| 172 | Vasopressin-regulated water reabsorption | 22 | 8 | 0 | 0.004550716 |
| 173 | Pancreatic secretion | 30 | 10 | 4 | 0.004579738 |
| 174 | Glycosaminoglycan degradation | 19 | 15 | 2 | 0.005369977 |
| 175 | Bacterial invasion of epithelial cells | 57 | 70 | 20 | 0.00553014 |
| 176 | Butanoate metabolism | 27 | 29 | 3 | 0.005921688 |
| 177 | Pancreatic cancer | 65 | 71 | 23 | 0.006543034 |
| 178 | Retrograde endocannabinoid signaling | 59 | 102 | 33 | 0.006739601 |
| 179 | Cysteine and methionine metabolism | 43 | 27 | 8 | 0.006822974 |
| 180 | Fatty acid degradation | 42 | 149 | 17 | 0.006822974 |
| 181 | Arginine biosynthesis | 21 | 8 | 3 | 0.007099497 |
| 182 | Nicotinate and nicotinamide metabolism | 29 | 36 | 2 | 0.007162293 |
| 183 | Morphine addiction | 54 | 78 | 30 | 0.00738237 |
| 184 | Staphylococcus aureus infection | 36 | 28 | 9 | 0.00919092 |
| 185 | Starch and sucrose metabolism | 36 | 23 | 15 | 0.00919092 |
| 186 | Tyrosine metabolism | 34 | 42 | 24 | 0.00919092 |
| 187 | Longevity regulating pathway - multiple species | 63 | 74 | 23 | 0.009463383 |
| 188 | Carbohydrate digestion and absorption | 18 | 16 | 1 | 0.011209123 |
| 189 | Fat digestion and absorption | 9 | 1 | 0 | 0.012460479 |
| 190 | Epithelial cell signaling in Helicobacter pylori infection | 37 | 19 | 6 | 0.014360189 |
| 191 | Transcriptional misregulation in cancer | 19 | 5 | 2 | 0.014781511 |
| 192 | Amino sugar and nucleotide sugar metabolism | 43 | 41 | 13 | 0.015083692 |
| 193 | Ether lipid metabolism | 44 | 65 | 9 | 0.015083692 |
| 194 | Vibrio cholerae infection | 16 | 4 | 3 | 0.017788119 |
| 195 | Pyruvate metabolism | 39 | 50 | 16 | 0.01836184 |
| 196 | Aminoacyl-tRNA biosynthesis | 13 | 14 | 3 | 0.020139563 |
| 197 | Selenocompound metabolism | 14 | 11 | 0 | 0.020139563 |
| 198 | Arrhythmogenic right ventricular cardiomyopathy (ARVC) | 10 | 7 | 1 | 0.020438241 |
| 199 | Fc epsilon RI signaling pathway | 61 | 68 | 22 | 0.020438241 |
| 200 | Glycolysis / Gluconeogenesis | 67 | 89 | 28 | 0.021647809 |
| 201 | Citrate cycle (TCA cycle) | 30 | 78 | 17 | 0.022216251 |
| 202 | Mucin type O-Glycan biosynthesis | 31 | 28 | 5 | 0.022216251 |
| 203 | Terpenoid backbone biosynthesis | 21 | 35 | 7 | 0.022965777 |
| 204 | Steroid hormone biosynthesis | 58 | 62 | 38 | 0.025443855 |
| 205 | mRNA surveillance pathway | 70 | 124 | 52 | 0.025986627 |
| 206 | Folate biosynthesis | 14 | 8 | 1 | 0.027381897 |
| 207 | Glutathione metabolism | 51 | 67 | 15 | 0.028989152 |
| 208 | Peroxisome | 8 | 4 | 0 | 0.028989152 |
| 209 | Propanoate metabolism | 32 | 58 | 15 | 0.028989152 |
| 210 | Pertussis | 52 | 67 | 25 | 0.031513183 |
| 211 | Alanine, aspartate and glutamate metabolism | 35 | 25 | 13 | 0.042230985 |
| 212 | Prion diseases | 20 | 10 | 1 | 0.044823525 |
| 213 | Pantothenate and CoA biosynthesis | 16 | 18 | 1 | 0.046683295 |
| 214 | Phenylalanine metabolism | 16 | 8 | 4 | 0.046683295 |

**Supplementary Table S4. The significant KEGG pathways identified by PoTRA for hepatitis B-induced HCC using the Kolmogorov–Smirnov test under the constructed network combining the correlation network and the pre-defined KEGG network.** FDR adjusted P-values are below 0.05. E.comb.normal represents the number of the combined network for normal samples, while E.comb.case represents that for cancer samples.
