# Supplementary material for: Pathways of topological rank analysis (PoTRA): a novel method to detect pathways involved in hepatocellular carcinoma
Source: PeerJ. 2018 Apr 9;6:e4571. doi: 10.7717/peerj.4571 (PMC5896492; doi:10.7717/peerj.4571)
Supplement: Table S5 — FDR adjusted P-values are below 0.05. E.comb.normal represents the number of the combined network for normal samples, while E.comb.case represents that for cancer samples. [file peerj-06-4571-s005.docx]

|  |  | Gene Counts | E.comb.normal | E.comb.case | Adjusted P value |
| --- | --- | --- | --- | --- | --- |
| 1 | Cytokine-cytokine receptor interaction | 253 | 125 | 63 | 0 |
| 2 | cAMP signaling pathway | 196 | 326 | 153 | 1.92713E-06 |
| 3 | MAPK signaling pathway | 252 | 451 | 171 | 6.26775E-06 |
| 4 | Pathways in cancer | 310 | 694 | 323 | 9.4804E-06 |
| 5 | alpha-Linolenic acid metabolism | 25 | 4 | 1 | 0.000122126 |
| 6 | Neuroactive ligand-receptor interaction | 28 | 5 | 2 | 0.000122126 |
| 7 | Allograft rejection | 28 | 4 | 3 | 0.000130972 |
| 8 | Calcium signaling pathway | 179 | 293 | 110 | 0.000130972 |
| 9 | Progesterone-mediated oocyte maturation | 89 | 90 | 37 | 0.000130972 |
| 10 | Taste transduction | 29 | 8 | 4 | 0.000195536 |
| 11 | Phototransduction | 27 | 6 | 3 | 0.00028085 |
| 12 | Oxidative phosphorylation | 47 | 86 | 11 | 0.000330908 |
| 13 | cGMP-PKG signaling pathway | 158 | 206 | 112 | 0.000820288 |
| 14 | NF-kappa B signaling pathway | 81 | 160 | 56 | 0.000820288 |
| 15 | Platinum drug resistance | 41 | 62 | 16 | 0.000820288 |
| 16 | Ovarian steroidogenesis | 39 | 43 | 9 | 0.000863906 |
| 17 | Adrenergic signaling in cardiomyocytes | 149 | 312 | 133 | 0.001121878 |
| 18 | Inflammatory mediator regulation of TRP channels | 91 | 105 | 46 | 0.001309432 |
| 19 | PI3K-Akt signaling pathway | 340 | 1536 | 615 | 0.001832855 |
| 20 | Oxytocin signaling pathway | 157 | 251 | 121 | 0.002347567 |
| 21 | p53 signaling pathway | 68 | 56 | 23 | 0.002539108 |
| 22 | Alcoholism | 167 | 481 | 439 | 0.002640944 |
| 23 | Aldosterone-regulated sodium reabsorption | 32 | 15 | 9 | 0.002640944 |
| 24 | Dilated cardiomyopathy | 76 | 181 | 88 | 0.002640944 |
| 25 | Ras signaling pathway | 226 | 638 | 238 | 0.002640944 |
| 26 | Tuberculosis | 173 | 324 | 140 | 0.002640944 |
| 27 | Tight junction | 125 | 221 | 73 | 0.002795935 |
| 28 | Epithelial cell signaling in Helicobacter pylori infection | 37 | 36 | 9 | 0.002974204 |
| 29 | mTOR signaling pathway | 144 | 289 | 111 | 0.0034778 |
| 30 | Salivary secretion | 48 | 41 | 17 | 0.004249112 |
| 31 | Apoptosis | 133 | 249 | 78 | 0.00435156 |
| 32 | Axon guidance | 167 | 308 | 148 | 0.00435156 |
| 33 | Hippo signaling pathway | 151 | 384 | 215 | 0.004446049 |
| 34 | Insulin secretion | 54 | 54 | 20 | 0.004446049 |
| 35 | Hepatitis C | 97 | 112 | 53 | 0.00464804 |
| 36 | VEGF signaling pathway | 61 | 105 | 28 | 0.00464804 |
| 37 | Drug metabolism - cytochrome P450 | 70 | 81 | 62 | 0.004764975 |
| 38 | Natural killer cell mediated cytotoxicity | 134 | 205 | 109 | 0.006278628 |
| 39 | Selenocompound metabolism | 14 | 16 | 2 | 0.006278628 |
| 40 | Regulation of actin cytoskeleton | 186 | 485 | 226 | 0.007493645 |
| 41 | Serotonergic synapse | 78 | 111 | 64 | 0.007493645 |
| 42 | Sphingolipid signaling pathway | 98 | 207 | 76 | 0.007493645 |
| 43 | Proteoglycans in cancer | 204 | 347 | 170 | 0.009447607 |
| 44 | Breast cancer | 143 | 253 | 126 | 0.009454863 |
| 45 | B cell receptor signaling pathway | 70 | 106 | 40 | 0.010706985 |
| 46 | Vascular smooth muscle contraction | 114 | 171 | 94 | 0.011311612 |
| 47 | Parkinson's disease | 29 | 18 | 5 | 0.013252798 |
| 48 | Insulin resistance | 94 | 139 | 54 | 0.014487325 |
| 49 | Measles | 102 | 141 | 73 | 0.014505916 |
| 50 | Chemokine signaling pathway | 187 | 841 | 387 | 0.014823051 |
| 51 | Gap junction | 88 | 116 | 63 | 0.015139881 |
| 52 | AMPK signaling pathway | 97 | 197 | 72 | 0.015257122 |
| 53 | Viral myocarditis | 26 | 16 | 4 | 0.015257122 |
| 54 | Protein processing in endoplasmic reticulum | 51 | 70 | 25 | 0.01528233 |
| 55 | Hepatitis B | 134 | 210 | 105 | 0.017555557 |
| 56 | Amyotrophic lateral sclerosis (ALS) | 36 | 21 | 5 | 0.018559452 |
| 57 | Toxoplasmosis | 93 | 111 | 53 | 0.019298727 |
| 58 | Type II diabetes mellitus | 47 | 43 | 16 | 0.019837159 |
| 59 | Toll-like receptor signaling pathway | 104 | 146 | 64 | 0.021151795 |
| 60 | N-Glycan biosynthesis | 49 | 110 | 54 | 0.021457254 |
| 61 | Nicotinate and nicotinamide metabolism | 29 | 87 | 15 | 0.021457254 |
| 62 | Maturity onset diabetes of the young | 24 | 6 | 9 | 0.021561208 |
| 63 | HTLV-I infection | 194 | 379 | 258 | 0.022117841 |
| 64 | Cell adhesion molecules (CAMs) | 94 | 67 | 41 | 0.023256813 |
| 65 | Lysine degradation | 55 | 117 | 58 | 0.023256813 |
| 66 | Amoebiasis | 44 | 37 | 15 | 0.023611696 |
| 67 | Amphetamine addiction | 62 | 98 | 55 | 0.023611696 |
| 68 | Small cell lung cancer | 83 | 194 | 90 | 0.023611696 |
| 69 | Renal cell carcinoma | 57 | 78 | 27 | 0.024042451 |
| 70 | Adherens junction | 71 | 133 | 63 | 0.027592607 |
| 71 | Herpes simplex infection | 104 | 146 | 81 | 0.029931242 |
| 72 | Glutamatergic synapse | 89 | 160 | 73 | 0.030678774 |
| 73 | Oocyte meiosis | 120 | 349 | 259 | 0.032303734 |
| 74 | NOD-like receptor signaling pathway | 48 | 89 | 41 | 0.034812057 |
| 75 | RIG-I-like receptor signaling pathway | 48 | 74 | 27 | 0.034812057 |
| 76 | Porphyrin and chlorophyll metabolism | 39 | 22 | 7 | 0.035620729 |
| 77 | Histidine metabolism | 23 | 11 | 6 | 0.036468617 |
| 78 | Rap1 signaling pathway | 208 | 589 | 236 | 0.036929314 |
| 79 | Glutathione metabolism | 51 | 144 | 23 | 0.037214316 |
| 80 | Glycerophospholipid metabolism | 94 | 448 | 104 | 0.037214316 |
| 81 | HIF-1 signaling pathway | 102 | 173 | 68 | 0.037214316 |
| 82 | Linoleic acid metabolism | 29 | 23 | 14 | 0.037214316 |
| 83 | mRNA surveillance pathway | 70 | 157 | 98 | 0.037214316 |
| 84 | Osteoclast differentiation | 123 | 299 | 213 | 0.037214316 |
| 85 | Renin secretion | 50 | 54 | 29 | 0.037214316 |
| 86 | Signaling pathways regulating pluripotency of stem cells | 112 | 204 | 102 | 0.037214316 |
| 87 | Thyroid hormone signaling pathway | 112 | 349 | 168 | 0.037214316 |
| 88 | Endocrine resistance | 95 | 189 | 82 | 0.037737607 |
| 89 | Bile secretion | 29 | 23 | 4 | 0.042146237 |
| 90 | Jak-STAT signaling pathway | 158 | 705 | 364 | 0.042146237 |
| 91 | Choline metabolism in cancer | 82 | 139 | 59 | 0.042231193 |
| 92 | Chronic myeloid leukemia | 73 | 154 | 75 | 0.042231193 |
| 93 | Cardiac muscle contraction | 13 | 4 | 0 | 0.042421633 |
| 94 | GABAergic synapse | 66 | 154 | 74 | 0.042566278 |
| 95 | AGE-RAGE signaling pathway in diabetic complications | 91 | 238 | 88 | 0.044670757 |
| 96 | Hypertrophic cardiomyopathy (HCM) | 25 | 12 | 8 | 0.044670757 |
| 97 | Melanogenesis | 101 | 158 | 83 | 0.046385334 |

**Supplementary Table S5. The significant KEGG pathways identified by PoTRA for alcohol-induced HCC using the Kolmogorov–Smirnov test under the constructed network combining the correlation network and the pre-defined KEGG network.** FDR adjusted P-values are below 0.05. E.comb.normal represents the number of the combined network for normal samples, while E.comb.case represents that for cancer samples.
